# The Impact of the COVID-19 Crisis on Air Travel Demand: Some Evidence From China

**DOI:** 10.1177/21582440231152444

**Published:** 2023-01-30

**Authors:** Xi Wu, Adam Blake

**Affiliations:** 1 Zhongyuan University of Technology, China; 2 Bournemouth University, Poole, UK

**Keywords:** COVID-19, air travel demand, demand recovery, baseline forecast, China

## Abstract

This paper applies a three-step framework to quantify COVID-19’s impacts on China’s domestic and international air travel demand and to evaluate how the impacts evolve from January 2020 to January 2022. Time series techniques and combination forecasting are tested to identify the best-performing model to generate baseline forecasts, with which actual demands are compared to assess the impact of COVID-19. The results demonstrate that during the period under study, China’s domestic aviation sector experienced two V-shape recoveries, while its international counterpart was devastated and showed no sign of revival. It suggests that to mitigate the impact of COVID-19, containing virus spread and removing mobility controls are essential; and when travel restrictions are lifted or loosened, governments play important roles in accelerating the rate of demand recovery.

## Introduction

The COVID-19 pandemic has caused substantial reductions in air travel demand both internationally and domestically. However, how much the impact is stays unknown. The objective of this paper is to quantify the impact of the COVID-19 outbreak on China’s air travel demand and evaluate how the impact evolves till January 2022. In addition, China’s experiences in promoting travel demand recovery are examined.

China was hit first by the virus and has made substantial progress in containing its spread. Differentiated measures based on each region’s epidemic situation have been implemented and most control measures have been removed in low-risk areas across mainland China, which is integral for travel recovery. On the other hand, international travels remain restricted by Chinese governments. Different pandemic situations in and outside China combined with different levels of restrictions regarding domestic and international travels result in sharp contrast in COVID-19’s impacts on China’s domestic and international aviation sector, which will be assessed and compared in this paper.

This paper contributes to the existing literature from three main aspects. Firstly, it quantifies the impact of the COVID-19 pandemic on air travel demand based on the latest available data till January 2022. The projected magnitude of the impact is vital to understand this unprecedented crisis. Secondly, this study compares COVID-19’s impacts on China’s domestic and international market and evaluates how and why the impacts change over time to explore the factors that affect the speed of demand recovery. The results provide valuable insights on how travelers behave during a tremendous health crisis. Moreover, this paper examines China’s experiences in reducing the damage of COVID-19. China has entered the cautious restart phase after the outbreak of the pandemic and has made notable achievements in promoting domestic travel demand recovery and driving economic growth, whose experiences will provide valuable information to the whole world.

The rest of this paper is organized into four sections. Section 2 reviews existing studies on the impacts of crises on travel/tourism demand. Section 3 discusses data and research method of this study with detailed introduction of the empirical strategy. The empirical results and discussion are reported in the subsequent section. And the conclusion is provided at last.

## Literature Review

In the current literature, there are mainly three approaches to assess the impacts of crises on travel/tourism demand quantitatively: introducing crisis-related variables which can represent the magnitude of crises into demand models; comparing baseline forecasts with actual demands; and generating scenario forecasts.

### Incorporating Crisis-Related Variables Into Demand Models

It is important to choose the “right” variable to represent the magnitude of crises, and traditionally, two types of variables are usually selected: dummy variables, which can specify the duration of crises; and the number of infections/infection rate or the number of deaths/mortality rate, which signals how severe health crises are.

It is a general practice to incorporate dummy variables into demand models to account for the effect of different types of one-off events, and the outbreaks of infectious diseases such as foot-and-mouth disease (FMD) and severe acute respiratory syndrome (SARS) are always considered (Cortes-Jimenez & Blake, 2011; Song et al., 2013; Zhang & Kulendran, 2016). However, the majority of such studies do not aim at evaluating the impacts of crises with only a few exceptions (Wang, 2009). However, dummy variables cannot be used to specify the duration of on-going crises which is still developing like the one that is torturing us.

Given available data on travel/tourism demand, the effect of both previous and on-gong health crises can be quantified through incorporating the number of infections/deaths into a demand model. For instance, Kuo et al. (2009) applied panel data models to assess the impact of avian flu and found significant impacts of the number of affected poultry outbreaks on global international tourism. Using a similar method, Kuo et al. (2008) found that the numbers of confirmed cases had significant impacts on SARS-infected markets but not on Avian flu- infected markets. Based on daily tourist arrivals data from January to April 2020, Tran et al. (2020) assessed the relationship between the number of confirmed cases and inbound tourism demand of Taiwan, Hong Kong, Thailand, and New Zealand and confirmed significant negative impact. They also suggested that destinations’ SARS experience affected the impact of the current pandemic.

When evaluating the impact of COVID-19 through such a method, new perspectives and new demand proxies are used. For example, Sharma and Nicolau (2020) adapted Karafifiath’s (1988) securities market-based approach to model the impact of COVID-19 on the market valuation of hotel, airline, cruise, and rental car industries using the threshold autoregressive conditional heteroskedasticity model (ARCH), where the number of infections and fatalities was introduced. They confirmed significant negative impact of COVID-19 on the market valuation of the four industries. Focusing on the impact of COVID-19 on tourists’ destination preferences in South Central China, X. Li et al. (2021) incorporated the difference in cumulative confirmed cases between destination and origin into panel data models where tourism demand was proxied with online sales of attraction tickets. They showed that Chinese domestic tourists avoided traveling to destinations with more confirmed cases than their origins.

In addition, new crisis-related variables are considered: some studies developed COVID-19 related indexes and others brought in big data. For example, Karabulut et al. (2020) developed the “Discussion about Pandemics Index,” a modified World Economic Uncertainty index (Ahir et al., 2020), to estimate the magnitude of pandemics and found that pandemics only decreased tourist arrivals to low-income countries. Yang et al. (2021) constructed the “COVID19 tourism index” to reflect tourism industry’s recovery process and to monitor the pandemic’s impacts on numerous aspects of tourism. Gallego and Font (2021) constructed leading indicators on future air travel demand using Skyscanner data on seat capacity, air passenger searches and picks and showed that big data could provide timely essential information regarding future demand during the COVID-19 pandemic. E. H. Wu et al. (2022) proposed a big data analytical framework using mixed data sampling models (MIDAS) to monitor and forecast hotel occupancy rates of Macau with search query data and found that MIDAS models could measure the dynamic impacts of the COVID-19 pandemic.

It is worth noting that big data can also be used to estimate the impact of COVID-19 without specifying demand models. For example, in the absence of actual passenger data, Sun et al. (2020) aggregated the global aviation sector into a system where flights connect each airport and country to evaluate the spatial-temporal evolution of international connectivity during the COVID-19 outbreak. They retrieved flight and airport data from Flightradar24 and OurAirports covering 150 airlines between 2,751 airports for 152 days to form worldwide airport, international country and domestic airport networks. They found that flight restrictions were mainly imposed on long-distance international flights and the evolutionary dynamics of domestic airport networks were closely correlated with the COVID-19 situation in specific countries. Focusing on China’s air passenger market, Warnock-Smith et al. (2021) assessed the impact of COVID-19 using both daily supply data from Official Airline Guide (OAG) and demand data from Sabre AirVision Market Intelligence Data Tapes (MIDT). They found that China’s air carriers and airports had been impacted unequally by the pandemic through an analysis of data on airline seats offered and passengers flown; airline revenues and average air fares; and airport frequencies.

### Comparing Baseline Forecasts With Actual Demands

Another way to assess the impacts of crises is to compare actual demands with demand forecasts which are generated on the assumption that crises had not happened. For instance, Page et al. (2012) proposed a method to separate the impacts of the 2008 global economic crisis and the swine flu pandemic, which overlapped with each other in time, on UK’s inbound tourism demand assuming that the swine flu pandemic was exogenous to economic growth. They applied the time varying parameter (TVP) technique to generate demand forecasts after producing predictions of explanatory variables using the exponential smoothing (ETS) approach, and the difference between the forecasts and the actuals was seen as the impact of the 2008 global economic crisis. To assess the impact of the swine flu pandemic, the actual values of the explanatory variables, which contained the information on economic crisis but not on the swine flu, were used to produce demand forecasts and the difference of these forecasts between the actual values of demand was considered as the impact of the swine flu. The COVID-19 pandemic is, however, not like the swine flu or the global economic crisis. It is endogenous to economic growth and its impact on travel/tourism demand is not sourced only from its economic influences but also from the imposition of control measures aiming at containing virus spread.

Data availability is a prerequisite for this approach: the impacts can only be assessed by comparing baseline forecasts with actual demands, on which data must be available. It is not popular to assess the impacts of on-going crises through such a method due to data limitation. Data on travel/tourism demand are always released quarters later and are always in low frequency (quarterly or yearly). However, the idea of generating baseline forecasts is also used to evaluate the impact of COVID-19 (Iacus, Natale, Santamaria, et al., 2020; Iacus, Natale, & Vespe, 2020; Kourentzes et al., 2021; Liu et al., 2021; Qiu et al., 2021; Zhang et al., 2021). For example, Iacus, Natale, and Vespe (2020) generated air traffic predictions through subtracting the expected reduction in air traffic, which was estimated considering air travel bans, from baseline demand forecasts, which were produced using a non-homogeneous Poisson process function based on historical data. The losses in passenger numbers due to observed route suppression were estimated based on real time online flight tracking platforms and on-line booking systems. They found that air passenger numbers from China dropped by 2.5% from January to March 2020 due to flight restrictions. The main limitation of their work is that they did not consider reductions in the number of passengers in still active routes/flights. The combination of tightened travel budget due to economic recession, the popularity of video conferencing, and consumers’ fear of catching the virus during flight leads to considerable losses in passenger numbers in active flights and routes, which should not be ignored.

### Scenario Forecasting

Building ex-ante demand forecasts based on different scenarios allows a range of possibilities to be identified. Usually, the “best,” “middle,” and “worst” case scenarios are set to reflect different levels of the severity of the crisis. For example, Smeral (2009, 2010) applied the error correction model (ECM) to evaluate the impact of the world recession and economic crisis on tourism. Ex-ante demand forecasts were projected in two different scenarios where the GDP loss due to the economic crisis was set at different levels. Similarly, the impacts of on-going crises can be investigated through generating future demand forecasts based on ex-ante projections of identified influencing factors. Song et al. (2011) and Song and Lin (2010) generated future demand forecasts based on ex-ante predictions of explanatory variables published by IMF and Euromonitor International to assess the impact of the 2008 global economic crisis.

Many studies incorporated the method of scenario forecasting to assess the impact of COVID-19 with different emphases. For instance, focusing on the effect of travel bans, Iacus, Natale, Santamaria, et al. (2020) predicted global air traffic till December 2020 and evaluated the resulted socio-economic impacts in different scenarios, which were set based on previous pandemic crisis and observed flight volume. According to their estimation, world GDP loss due to travel bans could be as high as 1.41% to 1.67% at the end of 2020 in the worst scenarios. Some studies applied the judgmental-adjusted scenario forecasting approach. Zhang et al. (2021) combined econometric and judgmental methods to forecast the possible paths of tourism recovery in Hong Kong based on different scenarios and concluded that demand for domestic and short-haul tourism would recover more rapidly than the long-haul markets. In the latest forecasting competition, Kourentzes et al. (2021), Liu et al. (2021), and Qiu et al. (2021) all applied the two-stage three judgmental-adjusted scenarios forecasting framework to evaluate the impact of COVID-19 on inbound tourism demand of 20 countries. They provided different tourism recovery rates in 2021 with the widest range from 36% to 77%.

In addition, different artificial intelligence techniques are also applied to generate forecasts in different scenarios. Polyzos et al. (2021) introduced the Long Short Term Memory (LSTM) neural network approach to estimate the impact of COVID-19 on China’s outbound tourism demand. They used data from SARS to train a single LSTM network and found that recovery to pre-crisis levels could take 6 to 12 months. Fotiadis et al. (2021) utilized both the LSTM algorithm and the Generalized Additive Model (GAM) to produce forecasts for international tourist arrivals in different scenarios, which were built based on data from three previous crises. They indicated that the drop in tourist arrivals could range between 30.8% and 76.3% and would persist at least until June 2021. Jaipuria et al. (2021) applied the artificial neural networks (ANN) to predict India’s tourist flows and foreign exchange earnings (FEE) in four scenarios and showed that if the tourism sector and policies were not restructured, FEE would fall below 1,790.53 million USD.

### Assessment of the Impacts of Crises on the Travel/Tourism Sector and the Whole Economy

When the impacts on the travel/tourism sector and the whole economy are considered, the general equilibrium model can be utilized. For example, Blake et al. (2003) incorporated the tourism sector into a Computed General Equilibrium (CGE) framework to measure the influence of the FMD outbreak on tourism and all other sectors of the UK economy and found that the FMD outbreak had larger adverse effects on GDP through reductions in tourism expenditures than through other effects. To assess the impact of COVID-19 on the whole economy, Yang et al. (2020) utilized the Dynamic Stochastic General Equilibrium (DSGE) model incorporating indicators of health disasters to demonstrate how tourism and the whole economy were affected by different infectious disease outbreaks. They provided simulated responses of the tourism sector in different scenarios with different levels of health disaster probability, disaster size, and disaster persistence. Pham et al. (2021) applied the tourism CGE model to estimate the economic impacts of the inbound tourism industry on the Australian economy during the pandemic in 2020. They argued that COVID-19 affected a range of industries and occupations beyond the tourism sector and called for strong support from the government on tourism.

After reviewing the current literature, it discovers that an increasing number of studies on the impact of COVID-19 on travel/tourism demand have emerged since 2020 with different research aims and perspectives. Many studies applied the scenario forecasting method to project the possible future. However, the quantification of the “historical” impact of COVID-19 is limited due to data limitation. Besides, the dynamic of COVID-19’s impact on air/travel demand and the possible drivers of the dynamic are under-studied. This paper complements existing studies as it quantifies the impacts of COVID-19 on China’s domestic and international air travel demand from January 2020 to January 2022, examines the dynamics of the magnitude of the impact and explores the factors that affect the recovery speed of China’s domestic air travel demand.

## Data and Research Method

### Data

In this study, air travel demand is proxied with passenger numbers. Monthly data on passenger numbers of Air China (AC), China Eastern (CE), and China Southern (CS) for both domestic and international routes are retrieved from China Stock Market & Accounting Research Database (CSMAR), a famous database in China. AC, CE, and CS are the largest three airlines in China, the market share of which were about 50% in 2019. The data spans the first 2 years of the COVID-19 outbreak and reaches up to January 2022. The starting and ending points of demand series vary from case to case dictated by data availability: the sample period is from 2006M01 to 2020M04 for AC; from 2006M01 to 2022M01 for CE; and from 2007M02 to 2022M01 for CS. For some cases where data are missing, the monthly values are computed based on the year-later values and the corresponding year over year (YOY) growth rates. The data is available upon request. The descriptive statistics for all variables are reported in Table 1.

**Table 1.** Descriptive Statistics.

Variable	Label	*N*	*M*	*SD*	Min	Max
Air China domestic	p_d_a	172	4,649.00	2,049.41	1,255.30	8,473.80
China Eastern domestic	p_d_e	193	5,500.24	2,234.12	1,005.03	9,996.50
China Southern domestic	p_d_s	180	7,238.00	2,185.73	1,415.19	12,247.14
Air China international	p_f_a	172	760.16	346.50	15.30	1,530.40
China Eastern international	p_f_e	193	711.08	435.49	15.02	1,600.96
China Southern international	p_f_s	180	717.53	499.99	29.65	1,969.65

### Empirical Strategy

This study applies a three-step approach to evaluate COVID-19’s impact through comparing baseline forecasts with actual demands.

#### Step 1: Identifying the Best Method to Generate Baseline Forecasts

Identifying a suitable model to generate accurate baseline forecasts is the first and an important step to assess COVID-19’s impact. In “normal” times, both causal econometric and non-causal time series techniques frequently appear in the current literature to forecast travel/tourism demand (Song et al. 2019). However, in the current situation, it is inappropriate to utilize econometric techniques, which require predictions of the independent variables before forecasting the dependent variable. The COVID-19 pandemic is endogenous to economic growth, and it is hard to predict its impact on economic factors. Just as Athanasopoulos et al. (2011) pointed out, the difficulty in forecasting the explanatory variables was one major reason of the unstable forecasting ability of causal econometric models. Besides, the impact of COVID-19 on air travel demand is not only conducted through its economic influences, but also through the imposition of control measures which restricts people’s mobility. So econometric models which only include economic influencing factors cannot capture the full scale of COVID-19’s impact.

In addition, in a series of studies, a variety of advanced forecasting methods including causal econometric and non-causal time series models, artificial intelligence methods, hybrid models, combination forecasting and hierarchical forecasting were compared to identify the best model to generate baseline forecasts. The empirical results suggested that univariate time series techniques, or combination and stacking model based on time-series models performed better than other forecasting methods in “normal” times according to both forecasting accuracy and robustness (Kourentzes et al., 2021; Liu et al., 2021; Qiu et al., 2021; Song & Li, 2021). As a result, three popular time series techniques including seasonal autoregressive integrated moving average (SARIMA), exponential smoothing (ETS), and state space ETS as well as combination forecasting based on them are chosen as candidate forecasting tools in this study, among which the best-performing method is identified and applied to generate baseline forecasts.

To compare the forecasting ability of the candidates, data ranging from 2006M01 to 2019M11 are used, which are divided into two periods: the model estimation period (2006M01/2007M02–2018M12) and the performance evaluation period (2019M01–2019M11). Forecasting performance is evaluated by mean absolute percentage error (MAPE) and root mean squared percentage error (RMSPE), which are commonly used in the current literature.

#### Step 2: Generating Baseline Forecasts and Measuring COVID-19’s Impacts

After the best forecasting method is identified, it is applied to generate baseline forecasts till 2022M01, which should represent the air travel demand that would have been recorded if the COVID-19 pandemic had not occurred. Actual values of demand are compared with the baseline forecasts and the impacts of COVID-19 are measured as the differences between them.

#### Step 3: Examining How COVID-19’s Impacts Evolve Till January 2022

The projected impacts of COVID-19 on domestic and international air travel demand are evaluated and compared to examine the dynamics of the impacts and the possible drivers of the dynamics.

### Candidate Forecasting Methods

#### Time Series Models

Non-causal time series models have a long history and wide applications in travel/tourism demand studies. The assumption underlying time series techniques is that travel/tourism demand can be modeled and forecast based on its own past values, which is justified by the belief that historic pattern of a time series can evolve into the future. As a result, the emphasis is put on revealing the historic trends and patterns (such as cycle and seasonality) of the series and predicting the future value of the series based on the properties identified (Song & Li, 2008). When forecasting tourism demand, there is no need to take the roles of explanatory variables into account, instead, the intrinsic evolution of tourism demand series is captured.

SARIMA, ETS, and state space ETS are popular time series techniques and have all been extensively applied as robust and powerful forecasting models (X. Wu, 2019). The ARIMA model was presented by Box and Jenkins in the 1970s and has become the most popular time series technique ever since (Goh & Law, 2011; Song & Li, 2008). It is renowned for its wide applicability, as it can handle any stationary or non-stationary time series, both with or without seasonality (Lim & McAleer, 2002). The SARIMA model is the most popular extension of the basic ARIMA model, which copes with seasonal data, and it has been proved to be a reliable forecasting tool when air travel demand is considered (Tsui & Balli, 2017; Tsui et al., 2014). The ETS model was developed based on the MA technique and uses weighted values of past observations to generate forecasts with the weights decaying exponentially over time justified by the belief that the most recent information is considered to be more influential on forecasts than older ones (X. Wu, 2019). It has been a popular technique for more than half a century and is simple to implement when forecasting data with seasonal patterns (Gounopoulos et al., 2012; Untong et al., 2015). The state space ETS model, which was proposed by Hyndman et al. (2002) encapsulates the notion of exponential smoothing in a state space form by including an observation equation for the forecast variable and a number of state equations for the components such as trend, level and seasonality which cannot be observed (X. Wu, 2019). It has been proved by many studies to have powerful forecasting ability (Gunter & Önder 2015; Hassani et al. 2017). Detailed discussions of SARIMA, ETS, and state space ETS methods can be found in Box et al. (2015), Holt (2004), and Hyndman et al. (2002), respectively.

#### Combination Forecasting

Combination forecasting produces composite forecasts by taking weighted averages of constituent predictions yielded by single models. The rationale is that single predictions from diverse models based on competing theories, functional forms, and specifications contain independent information, the combination of which can achieve diversification gain (X. Wu & Blake, 2022). The idea of combining multiple forecasts of the same event dates to the 1960s (Bates & Granger, 1969). Since then, the general forecasting literature has seen considerable studies on combination forecasts (Diebold & Pauly, 1990; Stock & Watson, 2004). Many travel/tourism demand studies showed that combining alternative forecasts together can reduce uncertainty and increase accuracy (G. Li et al., 2019; Shen et al. 2011; Wan& Song, 2018; J. Wu et al., 2020).

There exist different combination methods with the most popular one being the simple average (SA) method, which assigns equal weights to all individual forecasts (X. Wu & Blake, 2022). SA has been proved to be a robust, stable, and easy-to-use way, often outperforming more sophisticated combination methods, and hence is always used in combination forecasting studies (Makridakis & Winkler, 1983; Stock & Watson, 1998, 2003, 2004; J. Wu et al., 2020). Thus, this paper applies the SA method to combine SARIMA, ETS, and state space ETS. There are four different combinations referred to as combination 1 (COM1), combination 2 (COM2), combination 3 (COM3), and combination 4 (COM4), respectively, the components of which are shown in Table 2.

**Table 2.** Component Models of the Four Combinations.

	SARIMA	State space ETS	ETS
Combination 1 (COM1)	✓	✓	✓
Combination 2 (COM2)	✓	✓	
Combination 3 (COM3)	✓		✓
Combination 4 (COM4)		✓	✓

## Empirical Results and Discussion

### Performance Evaluation of Candidate Forecasting Models

Eviews 10.0 is used to generate forecasts for 2019M01 to 2019M11 based on data ranging from 2006M01 to 2018M12. The specific forms of the SARIMA model are selected based on Akaike Information Criteria (AIC) with automatic selections of the transformation form of the dependent variable. The maximum AR and MA orders are specified as 4 and the maximum SAR and SMA orders are specified as 1 with periodicity of 12 given monthly data are considered. The maximum differencing order is set as 2. The types of the error, trend, and seasonal components in the state space ETS models are automatically selected based on AIC, according to which the smoothing methods of the ETS models are chosen (X. Wu, 2019). Each route of each airline is treated, respectively, and the forecasting accuracy measures of each candidate model evaluated by MAPE and RMSPE are reported in Table 3 with the best results for each case highlighted.

**Table 3.** Forecasting Accuracy Measures (Evaluated for 2019M01–2019M11).

		SARIMA (%)	State space ETS (%)	ETS (%)	COM1 (%)	COM2 (%)	COM3 (%)	COM4 (%)
p_d_a	MAPE	2.21	5.57	8.27	5.16	3.60	4.95	6.92
	RMSPE	2.56	5.85	8.62	5.47	3.98	5.29	7.22
p_f_a	MAPE	5.10	4.97	4.93	4.92	4.96	4.94	4.95
	RMSPE	5.67	5.98	5.96	5.76	5.69	5.69	5.97
p_d_e	MAPE	2.37	4.00	5.51	3.22	2.33	2.90	4.76
	RMSPE	2.63	4.68	6.42	3.75	2.67	3.36	5.54
p_f_e	MAPE	1.71	3.07	3.48	2.50	2.09	2.22	3.26
	RMSPE	2.08	3.52	4.01	2.86	2.49	2.60	3.69
p_d_s	MAPE	1.84	1.95	2.22	1.69	1.58	1.95	1.94
	RMSPE	2.10	2.42	2.73	2.22	2.05	2.37	2.38
p_f_s	MAPE	1.91	2.43	3.23	2.13	2.07	2.41	2.40
	RMSPE	2.30	2.88	3.56	2.42	2.37	2.70	2.63

*Note*. Two decimal places are retained for all values.

Table 3 demonstrates that all candidate models perform well in forecasting domestic and international passenger numbers for all airlines with MAPEs and RMSPEs below 5% for most cases. However, the performance rankings vary from case to case. For forecasting international demand of AC and domestic demand of CE, MAPE, and RMSPE report controversial results regarding the most accurate model with RMSPE suggesting SARIMA and MAPE supporting COM1 and COM2, respectively. The combination models are chosen to produce baseline forecasts for these two cases mainly for two reasons. Firstly, the differences in RMSPEs of COM1 and COM2 and SARIMA are as small as 0.09% (between 5.76% and 5.67%) and 0.04% (between 2.67% and 2.63%), respectively. Secondly, combination forecasting represents the aggregation of various information embedded in different component models, which can be used in forecasting baselines. The models selected to generate baseline predictions for each case are summarized in Table 4.

**Table 4.** Models to Generate Baseline Forecasts for Each Case.

	Air China domestic (p_d_a)	Air China international (p_f_a)	China Eastern domestic (p_d_e)	China Eastern international (p_f_e)	China Southern domestic (p_d_s)	China Southern domestic (p_f_s)
Model	SARIMA	COM1	COM2	SARIMA	COM2	SARIMA

### The Impact on China’s Domestic and International Air Travel Demand

Baseline forecasts for 2019M12 to 2022M01, which represent the potential values of air travel demand that would have been recorded if the COVID-19 pandemic had not occurred, are generated for each case, respectively. The same forecasting method discussed in the previous section is applied.

#### The Domestic Market

**Figure 1.**
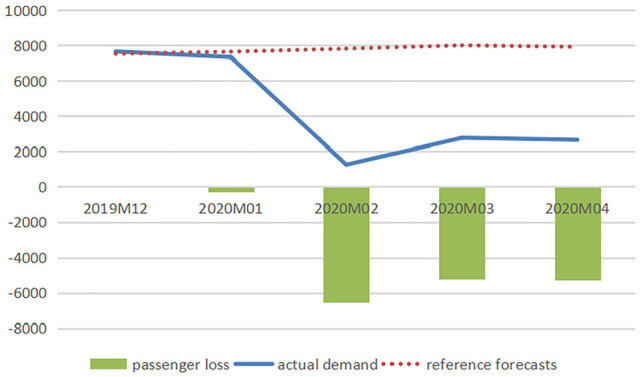
Baseline forecasts, actual values, and losses of domestic passenger numbers for air China (1,000 persons).

**Figure 2.**
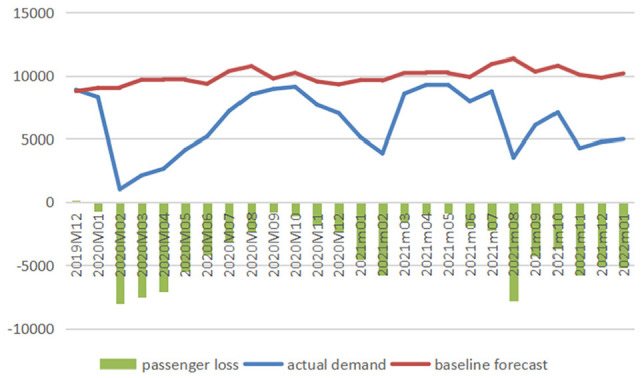
Baseline forecasts, actual values, and losses of domestic passenger numbers of China Eastern (1,000 persons).

**Figure 3.**
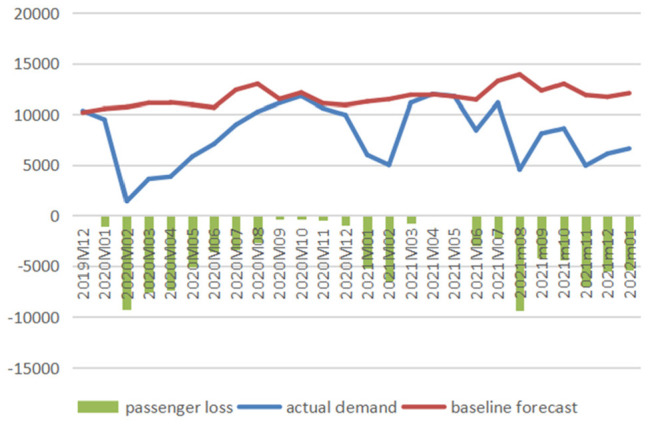
Baseline forecasts, actual values, and losses of domestic passenger numbers of China Southern (1,000 persons).

**Figure 4.**
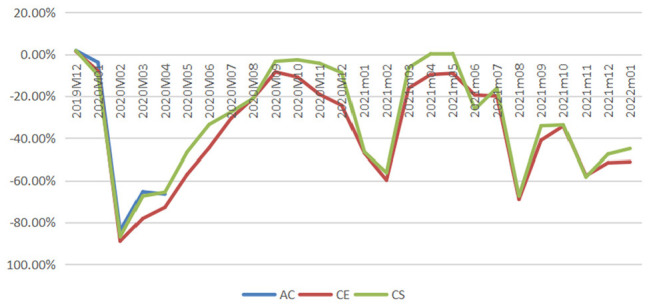
Percentage losses in domestic passenger numbers of air China (AC), China Eastern (CE), and China Southern (CS).

Figures 1 to 3 illustrate the comparison between baseline forecasts and actual values of the domestic passenger numbers of three airlines and report the impacts of COVID-19 on air travel demand measured in reductions in passenger numbers, and Figure 4 demonstrates the impacts measured in percentage losses generated from comparing reduced demands with baselines from December 2019 to January 2022.

The figures clearly show the significant losses of passenger numbers due to the pandemic with February 2020 being the hardest-hit month when AC, CE, and CS lost 6,553.82, 8,067.30, and 9,286.79 thousand passengers, respectively. The total losses in air traffic for AC from January to April 2020 are calculated to be 17,340.61 thousand persons (with total percentage loss calculated as 55.32%), and those for CE and CS from January 2020 to January 2022 are 94,525.09 thousand persons (total percentage loss: 37.82%) and 95,571.72 thousand persons (total percentage loss: 32.53%), respectively. There are 3 months (February–April) in 2020 and 1 month (August) in 2021 when the percentage losses exceeded 60%.

According to Figure 4, the magnitude of COVID-19’s impact changes dramatically over time during the period under study with three typical phases identified: two V-shape recovery phases (the first one from January to October 2020 and the second one from November 2020 to May 2021) and one unstable phase (from June 2021 to January 2022). In 2020, China’s domestic aviation sector witnessed the first rebound which emerged slowly but steadily after the bottom month (February 2020) and reached near the baseline in September 2020. The second revival emerged immediately after the trough in February 2021, with percentage losses reducing to 16.22% and 6.45% in the following month for CE and CS, respectively. In April and May 2021, CS even achieved surpluses with 0.15% and 0.4% more passengers recorded, respectively. During the unstable phase from June 2021 to January 2022, the magnitude of the impact fluctuated significantly with minor recoveries seen. For example, the percentage drop in air traffic for CS was 67.39% in August 2021 and it decreased to 34.16% 1 month later.

#### The International Market

**Figure 5.**
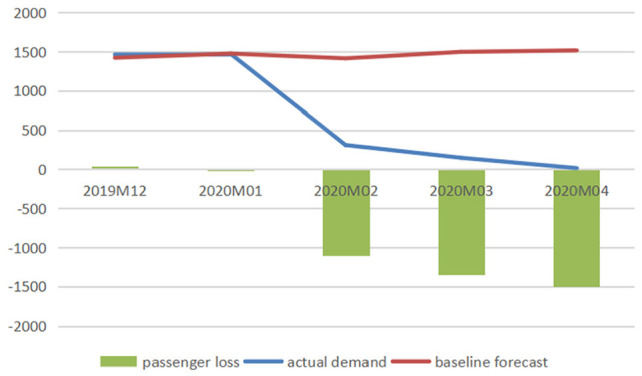
Baseline forecasts, actual values, and losses of international passenger numbers for air China (1,000 persons).

**Figure 6.**
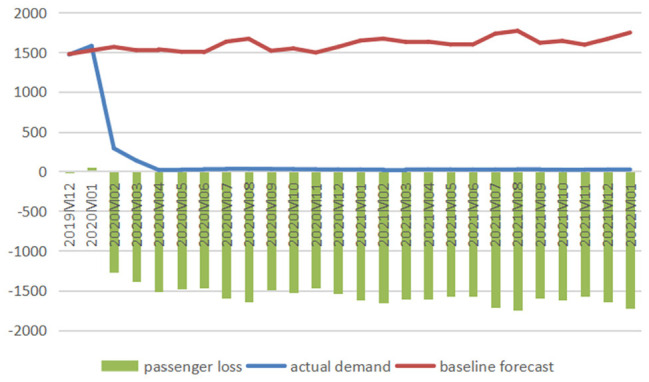
Baseline forecasts, actual values, and losses of international passenger numbers for China Eastern (1,000 persons).

**Figure 7.**
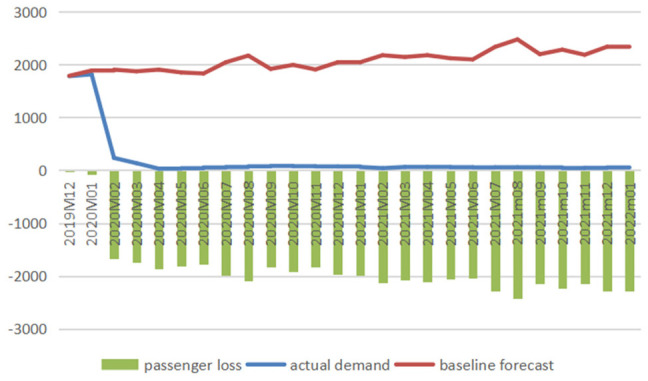
Baseline forecasts, actual values, and losses of international passenger numbers for China Southern (1,000 persons).

**Figure 8.**
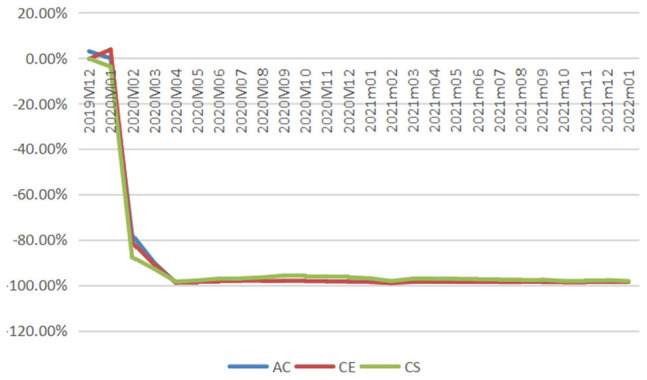
Percentage losses in international passenger numbers of air China (AC), China Eastern (CE), and China Southern (CS).

Figures 5 to 7 illustrate the comparison between baseline forecasts and actual values of international air traffic of three airlines and present the impacts of COVID-19 measured in reductions in passenger numbers, and Figure 8 shows the impacts measured in percentage losses obtained from comparing reduced demands with baselines from December 2019 to January 2022.

The figures clearly demonstrate the devastating effect of COVID-19 on China’s international aviation sector, which has been hit hard since February 2020. Passenger numbers of AC, CE, and CS all experienced sharp drops in February 2020, and the downward trend carried over through March to April 2020, when air traffic dropped to a minimal level and stayed there till January 2022. In April 2020, the COVID-19 crisis induced 1,498.96, 1,517.07, and 1,874.07 thousand passengers lost, which were almost the full potential of international demand, dragging the actual demands to 15.30, 17.60, and 29.65 thousand persons for AC, CE, and CS, respectively. After hitting bottom in April 2020, China’s international air travel demand showed no sign of improvement with considerable reductions in passenger numbers till January 2022. As shown in Figure 8, the percentage losses stayed above 98% for CE and above 95% for CS from April 2020 to January 2022. The total losses in air traffic are calculated to be 3,950.99 thousand persons (total percentage loss: 89.34%) for AC from February to April 2020, 37,632.35 thousand persons (total percentage loss: 97.54%), and 48,740.19 thousand persons (total percentage loss: 96.82%) for CE and CS, respectively, from February 2020 to January 2022.

### The Dynamics of COVID-19’s Impacts

**Figure 9.**
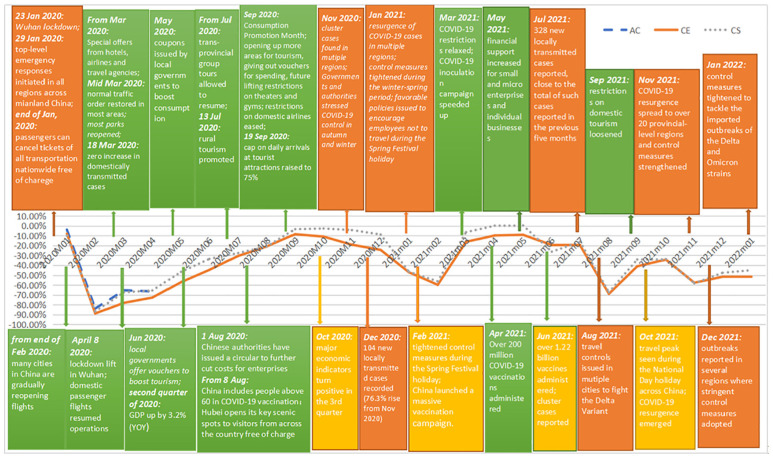
Dynamics of COVID-19’s impacts on China’s domestic air travel demand and the timeline of the COVID-19 crisis in China.

**Figure 10.**
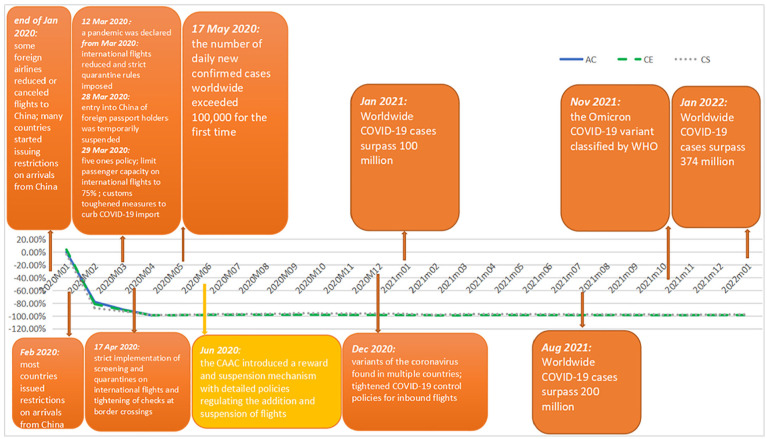
Dynamics of COVID-19’s impacts on China’s international air travel demand and the timeline of the COVID-19 crisis worldwide.

The contrast between the magnitude of COVID-19’s impacts on China’s domestic and international air travel demand is sharp. China’s domestic civil aviation industry experienced two major V-shape recoveries, its international counterpart, however, had suffered from the crisis since February 2020 and had shown no signal of revival till January 2022. Figure 9 integrates the timeline of the COVID-19 pandemic in China and the figure illustrating the dynamics of COVID-19’s impacts on China’s domestic air travel demand, which is measured by percentage losses in passenger numbers of the three carriers; and Figure 10 integrates the international counterparts. The information regarding the timeline of the pandemic is collected from the official website of the State Council of China (http://english.www.gov.cn/news/).

Upon observation of Figures 9 and 10, it reveals that the pandemic situation and the corresponding restriction level fundamentally determine the magnitude of the impact. The imposition of control measures which is triggered by the spread of the virus has instant negative effects on air travel demand both domestically and internationally. And the magnitude of the effect is determined by the scale and intensity of the control. National or international control measures resulted from severe and complicated pandemic situations can generate instantaneous, dramatic, and long-lasting damage. For China’s domestic market, passenger numbers plummeted by about 86% in February 2020 following top-level emergency responses activated in all regions in mainland China on 29 January 2020. Mobility restrictions were generally lifted since the end of February 2020, nevertheless, domestic air traffic only restored no more than 35% of its potential by April 2020. A nearly full recovery took 7 months from March to September 2020 to achieve, when CE and CS won back 91.5% and 96.57% of their potential customers, respectively.

Regarding the international market, air traffic dropped by about 80% in February 2020 as some foreign airlines started to reduce or cancel flights to China from the end of January 2020 and most countries issued restrictions on arrivals from China in February 2020. The situation in the international market deteriorated in March and April 2020 as China imposed strict restrictions on international flights under the pressure of containing the risks of imported COVID-19 cases given the explosive growth of infections worldwide. The combination of the Five Ones police, the passenger capacity limit, the strict quarantine rules, and the health certificate requirement produced notable results dragging international passenger numbers to a minimal level in April 2020, which last till January 2022.

Comparatively, the effect of regional control measures resulted from milder COVID-19 situation is less and briefer. For instance, China tightened travel controls in medium and high-risk areas in November 2020 after cluster cases reported in some regions, and CE and CS lost 19.21% and 4.36% potential customers that month. The marked demand drop (by more than 56%) in February 2021 was fueled by a variety of reginal control measures in four consecutive months (from November 2020 to February 2021): people living in low-risk areas were advised to avoid travel unless necessary; tourist attractions were required to operate under limited capacity and reservation policies; and a plan was issued to reduce mass gatherings and people’s movements during the 2021 Spring Festival travel rush. According to a survey which covered 53,107 migrant workers from around 500 enterprises, over 77% of migrant workers chose not to go home for Chinese New Year in 2021 (Xinhua, 2021a). Impressively, a nearly full recovery only took 1 month to achieve after the restrictions were relaxed from March 2021. The percentage losses in air traffic were 59.86% and 56.37% in February 2021 for CE and CS, respectively, and the reductions dropped to 16.22% and 6.45% in March 2021.

Figure 9 also shows that although recoveries were seen during the unstable period from June 2021 to January 2022, full demand restoration failed to emerge mainly due to the resurgences of COVID-19 cases and the resulted tightened control measures. From the end of August 2021, domestic demand started to revive smoothly after fighting the Delta variant for 2 months until a new wave of COVID-19 outbreaks recorded from mid-October 2021. From November 2021 to January 2022, percentage demand losses are projected to stay above 45% under the pressure to tackle the Delta and Omicron strains with stringent control measures adopted in medium- and high-risk regions.

### China’s Experiences in Promoting Travel Demand Recovery

According to Figure 9, China has made notable achievements in promoting travel demand recovery during the COVID-19 pandemic. Obviously, containing virus spread and removing mobility controls are essential for travel demand recovery. Moreover, China’s experiences in accelerating the speed of demand recovery can be summarized into two aspects.

Firstly, the implementation of promotion campaigns and preferential policies, which can reduce the cost of travel and support employment, is important in mitigating the impact of COVID-19, which does not only transmit through the imposition of mobility restrictions but also through its influential economic consequences. The economy is hit hard by the crisis bringing about unemployment, bankruptcies, and general depression. With tightened budget, peoples’ intention to travel declines. Consequently, strong support from the government is critical. During 2020’s summer holiday, the average price of flights in China was 30% lower than that of the previous summer and hotels offered sales with average savings of 25% (Cheng, 2020). Besides, local governments issued coupons to boost consumption. As of 26 April, 2020, 11.5 billion RMB worth of consumption coupons had been issued in 25 provinces and municipalities (Wang, 2020). In addition, preferential policies have been implemented to support employment and sectors that are hit hard by the pandemic. For example, the loss carryforward period for sectors including transportation, catering, and tourism was extended from 5 to 8 years (Xinhua, 2020). As shown in Figure 9, these policy responses drove up the demand for domestic air travel, which gradually revived to about 95% of its potential in September and October 2020.

Secondly, the restoration of consumers’ sense of security through normalizing epidemic prevention measures and initiating vaccination campaign is necessary to mitigate the impact of the pandemic. During a pandemic, most consumers have health concerns worrying that they may catch the virus when they travel and consequently are unwilling to travel even after the epidemic has been put under control. This is one important reason why air travel demand was not as sensitive to the lift of restrictions as to the imposition of them during the first V-shape recovery phase. Figure 9 shows that the relaxation of control measures beginning at the end of February 2020 failed to induce instant significant recovery, instead, it took 7 months to achieve a nearly full revival. As a result, appropriate measures are necessary to ensure peoples’ health and safety.

In China, epidemic prevention has become the new normal with protection measures such as wearing masks and keeping social distance becoming a part of peoples’ everyday life. Differentiated measures to curb virus transmission based on each region’s situation have been taken since the end of February 2020 with standardized measures enforced throughout the travel process, which have been proved to be effective in combating virus spread. In addition, China has launched a massive vaccination campaign from the end of February 2021 to provide free COVID-19 vaccination to Chinese residents. By 25 May 2021, more than 546.71 million doses of COVID-19 vaccines had been administered across China (Xinhua, 2021b). With all these efforts, peoples’ health concerns faded gradually which induced more travel demand. Figure 9 demonstrates that the recovery speed in the second V-shape recovery phase quickened impressively with significant recovery emerging as soon as mobility controls were loosened in March 2021. It means that consumers were more willing to travel than a year ago as long as they could, which implies that consumers’ sense of security was restored.

## Conclusion

This paper quantifies COVID-19’s impacts on China’s domestic and international air travel demand till January 2022, examines the dynamics of the magnitude of the impacts and explores the factors that affect the recovery speed of China’s domestic air travel demand. The empirical results clearly show the considerable damage caused by COVID-19 to China’s civil aviation sector with the domestic market witnessing two V-shape recoveries and the international market being devastated with no sign of revival. It demonstrates that the magnitude of COVID-19’s impacts is mainly determined by the pandemic situation and the corresponding scale and intensity of controls. Besides, when travel restrictions are lifted or loosened, governments play important roles in accelerating the rate of demand recovery.

The results of our study are instructive. It proves that travel demand revival can be achieved while containing the COVID-19 outbreak as long as appropriate strategies are taken. According to Figure 9, a nearly full recovery in domestic air travel demand took 7 months in 2020 and 1 month in 2021 to emerge. From the experiences of China, the capacity for travel demand revival is fundamentally determined by the pandemic situation and the corresponding restriction level, but they are not the only factors that determine whether people will travel again. The speed of travel demand recovery can be accelerated significantly if appropriate measures are taken. The difference in the projected speeds of the revival in China’s domestic air travel demand between the first and the second V-shape recovery phases shows the importance of governments’ roles in mitigating the impact of COVID-19 through promoting consumption and restoring consumers’ sense of security.

Our analysis is not free of limitations. Econometric models are excluded in the first step of our research as accurate forecasts for explanatory variables such as travelers’ disposable income or consumer price index are not available. For future research, if reliable predictions of such inputs are available, econometric models can also be tested when identifying the best model to generate baseline forecasts. Besides, aggregate air travel demand is not studied due to data limitation. When data on aggregate demand is available, the same research framework can be followed to evaluate the impact of COVID-19 on aggregate travel demand.
